# Effectiveness of Pemafibrate Dose Escalation on Metabolic Dysfunction-Associated Steatotic Liver Disease Refractory to Standard Dose

**DOI:** 10.3390/metabo15020100

**Published:** 2025-02-05

**Authors:** Satoshi Shinozaki, Kouichi Miura, Toshiyuki Tahara, Hironori Yamamoto

**Affiliations:** 1 Shinozaki Medical Clinic, Utsunomiya 321-3223, Japan; 2 Department of Medicine, Division of Gastroenterology, Jichi Medical University, Shimotsuke 329-0431, Japan; 3 Saiseikai Utsunomiya Hospital, Utsunomiya 321-0974, Japan

**Keywords:** non-alcoholic fatty liver disease, non-alcoholic steatohepatitis, pemafibrate, dyslipidemia, metabolic dysfunction-associated steatotic liver disease

## Abstract

**Background and Aim:** Controlling the hepatic inflammation of metabolic dysfunction-associated steatotic liver disease (MASLD) is important to prevent serious condition. Pemafibrate, a selective peroxisome proliferator-activated receptor-α modulator, has demonstrated effectiveness at a standard dose (0.2 mg daily). The aim of this study is to evaluate the effectiveness of pemafibrate dose escalation from 0.2 mg to 0.4 mg daily in patients with MASLD who are refractory to standard-dose therapy. **Methods:** This study included patients with MASLD who had a persistent elevation of alanine aminotransferase (ALT) levels despite more than one year of standard-dose pemafibrate therapy (0.2 mg daily). All patients underwent dose escalation to 0.4 mg once daily. Hepatic inflammation was assessed using serum ALT levels, hepatic function was evaluated with the albumin–bilirubin score, and hepatic fibrosis was estimated using Mac-2 binding protein glycosylation isomer (M2BPGi) levels. A one-year treatment period was investigated, including six months before dose escalation and six months after dose escalation. **Results:** Eleven patients were included. The median treating period with standard-dose pemafibrate was 3.2 years. Weight did not show significant change throughout the observation period. Regarding the hepatobiliary enzyme, the aspartate aminotransferase, ALT, and γ-glutamyl transpeptidase levels significantly improved six months after the dose escalation. Specifically, ALT improved in all patients, and the ALT levels normalized in four patients (36%). The lipid profiles, the albumin–bilirubin score, and M2BPGi did not significantly change after the dose escalation. **Conclusions:** The dose escalation of pemafibrate from 0.2 mg to 0.4 mg daily may improve hepatic inflammation in patients with MASLD refractory to standard-dose therapy.

## 1. Introduction

Excessive fat accumulation of the liver is the hallmark of metabolic dysfunction-associated steatotic liver disease (MASLD). Persistent hepatic inflammation can lead to metabolic dysfunction-associated steatohepatitis (MASH), which may progress to cirrhosis and hepatocellular carcinoma. The prevalence of MASLD is increasing worldwide [1], and it is associated with obesity and insulin resistance. The interaction between the liver, adipose tissue, intestines, and other organs contributes to the progression of MASLD/MASH. This involves oxidative stress due to increased lipid influx into hepatocytes, increased insulin resistance, abnormal adipokine secretion from adipose tissue, and endotoxin translocation from the intestines, all of which are implicated in the onset and progression of MASLD/MASH [2]. To prevent these unfavorable progressions, the long-term control of hepatic inflammation in MASLD is important. Recently, a selective thyroid hormone receptor-β agonist, resmetirom, was approved by the U.S. Food and Drug Administration (FDA) in March 2024 for the treatment of MASLD. However, few medications are formally approved for MASLD therapy.

Pemafibrate, a selective peroxisome proliferator-activated receptor-α modulator (SPPARMα), was approved in Japan as a medication for dyslipidemia in 2018. We reported the 3-month and 1-year effectiveness of standard-dose pemafibrate 0.2 mg daily in patients with both dyslipidemia and MASLD [3,4]. Improvements in hepatobiliary enzyme with pemafibrate on MASLD patients were also confirmed by a recent systematic review that reported an approximate 35 U/L reduction in alanine aminotransferase (ALT) on average within six months [5]. A phase II trial demonstrated that pemafibrate had a significant effect on magnetic resonance elastography (MRE)-based liver stiffness over 72 weeks [6]. A large randomized–controlled trial revealed a significant reduction in MASLD incidence in the pemafibrate group compared to the placebo group by 22% (hazard ratio 0.78, 95% confidence interval 0.63–0.96, *p* = 0.02) [7]. Given the dose-dependent pharmacological effects of pemafibrate, dose escalation may offer additional therapeutic benefits. However, there are no reports on the effectiveness of the dose escalation of pemafibrate in refractory MASLD. The aim of this study is to clarify the effectiveness of the dose escalation of pemafibrate from 0.2 to 0.4 mg daily in patients with MASLD who had been refractory to pemafibrate 0.2 mg daily.

## 2. Patients and Methods

### 2.1. Study Population

This retrospective review included patients who underwent dose escalation of pemafibrate from 0.2 to 0.4 mg daily to improve refractory MASLD. Medical records and laboratory findings for the one-year treatment period were investigated, including six months before the dose escalation and six months after the dose escalation.

The inclusion criteria were as follows: (1) steatotic liver disease diagnosed by ultrasound; (2) presence of dyslipidemia that met cardiometabolic criteria [8]; (3) sustained elevation of ALT >30 for more than six months despite standard-dose pemafibrate therapy (0.2 mg daily) for more than one year; (4) dose escalation to 0.4 mg once daily was performed; (5) follow-up data were available for more than six months after the dose escalation; (6) negative hepatitis B surface antigen and hepatitis C virus antibody tests; (7) normal serum immunoglobulin-G level; and (8) alcohol consumption <30 g/day in males and <20 g/day in females [4]. Medication adherence was estimated by the frequency of clinic visits and records of prescription. Regular attendance at scheduled appointments was considered indicative of good adherence to the prescribed pemafibrate regimen. The Institutional Review Board of the Shinozaki Medical Clinic approved this retrospective review on 25 October 2024 (ID#06-R001), and this retrospective observational study was exempt from the requirement for individual consent.

### 2.2. Evaluation of Hepatic Markers

To evaluate hepatic inflammation, we assessed the serum ALT level. The American Association for the Study of Liver Diseases (AASLD) practice guidance states that ALT normalization can predict metabolic dysfunction-associated steatohepatitis resolution, and ALT > 30 U/L should be considered abnormal [9]. We used the albumin–bilirubin (ALBI) score calculated with serum albumin and total bilirubin levels to assess hepatic function. The ALBI score correlates with the indocyanine green retention test at 15 min [10]. The degree of hepatic fibrosis was estimated using the Mac-2 binding protein glycosylation isomer (M2BPGi) [11].

### 2.3. Statistical Analysis

Changes in parameters from baseline to six months after dose escalation were assessed using the Wilcoxon signed-rank test. Comparisons of parameters at multiple time points were evaluated using the Friedman test. These analyses were performed using the BellCurve for Excel (Social Survey Research Information Co., Ltd., Tokyo, Japan). Differences were considered significant with *p* < 0.05.

## 3. Results

### 3.1. Baseline Characteristics

The baseline characteristics of the 11 patients who fulfilled the inclusion criteria are shown in Table 1. Approximately half of the patients were treated with statins. The median treating period with the standard dose was 3.2 years. There were no unexpected dose changes during the study period. Medication adherence was high, with an estimated rate of over 95% throughout the study period.

### 3.2. Changes in Parameters Before and After Dose Escalation

**Figure 1:**
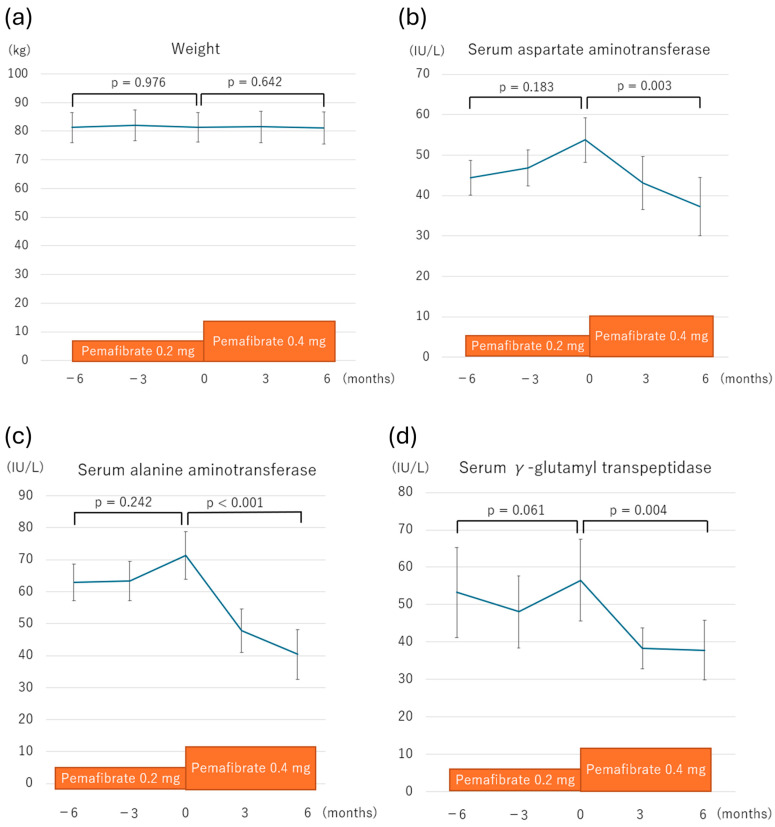
Changes in key parameters over the six months before and after dose escalation of pemafibrate from 0.2 mg to 0.4 mg daily. Panels depict the following parameters: (**a**) weight, (**b**) aspartate aminotransferase (AST), (**c**) alanine aminotransferase (ALT), and (**d**) γ-glutamyl transpeptidase (γ-GTP). “Before dose escalation” represents data at 6 and 3 months prior to the escalation. “After dose escalation” represents data at 3 and 6 months following the escalation. Statistical significance was assessed using the Friedman test. Error bars indicate the standard error of the mean.

The weight and laboratory findings for the one-year treatment period, including six months before the dose escalation and six months after the dose escalation, are shown in Table 2. Weight did not show a significant change throughout the observation period. Regarding the hepatobiliary enzyme, the aspartate aminotransferase (AST), ALT, and γ-glutamyl transpeptidase (γ-GTP) levels significantly improved six months after the dose escalation (Figure 1). Specifically, ALT, a marker of hepatic inflammation, improved in all patients with an average reduction of 30.9 IU/L (Supplementary Figure S1). The ALT levels normalized in four patients (36%). The lipid profile (LDL cholesterol, HDL cholesterol, triglycerides), a marker of hepatic function (the ALBI score), and a marker of hepatic fibrosis (M2BPGi) did not significantly change after the dose escalation.

Regarding safety, in addition to weight, we reviewed the medical records for any reported adverse events, changes in laboratory findings such as renal function (estimated GFR), and clinical symptoms during follow-up. No adverse events or unexpected side effects were observed.

## 4. Discussion

We have demonstrated that the dose escalation of pemafibrate is effective in improving hepatic inflammation in patients with refractory MASLD. Additionally, all patients in the present study responded to the dose escalation. Although many reports have shown the effectiveness of pemafibrate at the standard dose, this is the first study demonstrating the effectiveness of dose escalation. Therefore, the dose escalation of pemafibrate is a viable option for refractory MASLD.

Obesity (BMI > 30) is a factor that can cause refractory to standard-dose pemafibrate therapy, as we previously reported [12]. Indeed, the baseline BMI and weight of patients who entered the present study were approximately 30 and 80 kg, respectively. These findings differed from the characteristics of patients with MASLD who enrolled in our previous study (BMI of 27 and weight of 70 kg on average) [4], in which standard-dose pemafibrate was used. The reason why standard-dose pemafibrate failed to decrease transaminases in patients with obese MASLD is largely unknown. Body weight may affect the effectiveness of standard-dose pemafibrate on serum transaminase levels. Pemafibrate did not change the total volume of hepatic fat in an animal model [13] or in humans [6]. However, pemafibrate can decrease the size of lipid droplet in hepatocytes [13], which is a potential mechanism by which pemafibrate decreases serum transaminases in patients with MASLD. Double-dose pemafibrate therapy (0.4 mg daily) may have further decreased the size of lipid droplets in the liver.

We have previously reported that standard-dose pemafibrate therapy had favorable effects on hepatic function and fibrosis [3,4]. However, the dose escalation did not show significant improvements in hepatic function and fibrosis. There are a few suspected reasons for this. First, the dose escalation in the present study did not lead to further improvements in cardiometabolic factors, including dyslipidemia and diabetic parameters. Cardiometabolic factors can contribute to hepatic function and fibrosis [14]. Thus, additional treatments are required for further improvements in hepatic function and fibrosis. A recent meta-analysis demonstrated the effectiveness of sodium–glucose cotransporter-2 (SGLT2) inhibitors in decreasing total fat volume [15]. The combined use of SGLT2 inhibitors and double-dose pemafibrate may enhance amelioration by decreasing the total amount of liver fat and size of lipid droplets [16]. Second, differences in patients’ characteristics may have led to these results. Unlike previous studies involving naïve subjects, the patients enrolled in the present study were pemafibrate-treated individuals. Therefore, the initial treatment with pemafibrate may have already had the maximum effect on these parameters.

Few articles are available regarding the effectiveness of double-dose pemafibrate therapy on MASLD regardless of the included population. A post hoc analysis including six phase II and phase III randomized, double-blind, placebo-controlled trials reported mild superiority of double-dose pemafibrate (0.4 mg daily) compared to standard-dose pemafibrate (0.2 mg daily) in the improvement in AST, ALT, and γ-GTP [17]. However, more than half of the patients included in this post hoc analysis did not have steatotic liver disease, and no significant differences were observed between the standard-dose and double-dose groups. Therefore, we are the first to evaluate the effectiveness of double-dose pemafibrate therapy on MASLD.

The biosafety of pemafibrate at a double dose (0.4 mg daily) has been previously demonstrated in several studies, including phase II and phase III clinical trials. Yokote et al. conducted a pooled analysis of six randomized, double-blind, placebo-controlled trials, which included patients treated with both the standard dose (0.2 mg daily) and double dose (0.4 mg daily). The analysis reported no significant increase in adverse events between the two groups, suggesting that the double dose is well tolerated [17]. Additionally, in our current study, we observed no adverse events or unexpected dose-related side effects in the 11 patients included, further supporting the biosafety of the double dose in clinical practice. However, we acknowledge the importance of ongoing monitoring to detect any rare or long-term risks associated with higher doses of pemafibrate.

The strength of this study is the data availability of data before and after the dose escalation. In fact, refractoriness was demonstrated by patients who had undergone more than one year of standard-dose pemafibrate therapy and a six-month observation period before starting the dose escalation. Moreover, the estimated adherence rate exceeded 95%, indicating that patients consistently followed the prescribed pemafibrate regimen. This high level of adherence strengthens the association between dose escalation and the observed improvements in hepatic enzymes. The limitations are as follows: (1) this is a single-center retrospective observational study without control groups, and (2) we combined the use of statin or SGLT2 inhibitor. While these medications may contribute to overall metabolic and hepatic health, their specific impact on the outcomes observed in this study was not separately evaluated due to the small sample size, and (3) there was no histopathological assessment of the liver.

In conclusion, the dose escalation of pemafibrate may be effective for improvement in hepatic inflammation in patients with MASLD that is refractory to standard-dose pemafibrate therapy. However, improvement in lipid profiles or hepatic fibrosis may not be expected within six months. Larger studies with extended follow-up periods are needed to validate these preliminary findings.

## Figures and Tables

**Table 1:** Baseline characteristics.

	n = 11
Age, years, median (IQR)	45 (41–48)
Gender, male, n	8 (73%)
Current smoker, n	0 (0%)
Complications treated with medications, n	
Hypertension	5 (45%)
Gastroesophageal reflux disease	2 (18%)
Diabetes mellitus	1 (9%)
Concurrent medications, n	
Statins	5 (45%)
Angiotensin II receptor blocker	3 (27%)
Sodium–glucose cotransporter-2 inhibitor	1 (9%)
Interval from starting standard dose of pemafibrate to dose escalation, year, median (IQR)	3.2 (1.9–3.7)

IQR: interquartile range.

**Table 2:** Changes in clinical parameters before and after six months of dose escalation.

	Six Months Before	Baseline	Six Months Later	*p*-Value *
Weight, kg, median (IQR)	82 (71–87)	80 (71–87)	80 (69–88)	0.575
Body mass index	29.7 (27.9–30.8)	29.6 (28.2–31.0)	29.6 (27.5–30.9)	0.505
AST, U/L	41 (37–56.5)	57 (38–69)	30 (22.5–35.5)	0.016
ALT, U/L	62 (50–74.5)	58 (55–83)	33 (22.5–38)	0.003
γ-GTP, U/L	43 (33.5–56)	50 (37.5–60.5)	32 (23.5–49)	0.007
Platelet count, ×10^4^/μL	27.4 (24.6–30.9)	27.2 (23.4–31.2)	29 (23.3–30.9)	0.755
Estimated GFR, mL/min/1.73 m^2^	75.3 (73.2–88.8)	77.7 (70.5–82)	75.8 (74.2–81.1)	0.755
LDL cholesterol, mg/dL	94 (92–102)	93 (83–102)	85 (77–97)	0.449
HDL cholesterol, mg/dL	44 (39–50)	43 (39–53)	48 (38–54)	0.798
Triglyceride, mg/dL	103 (69–152)	97 (81–115)	84 (64–112)	0.476
Uric acid, mg/dL	5.8 (4.3–7.0)	6.0 (4.3–6.8)	5.8 (4.9–6.8)	0.858
Fasting plasma glucose, mg/dL	109 (100–130)	106 (93–112)	109 (103–128)	0.130
Serum insulin, µU/mL	12.2 (8.7–16.5)	9.4 (6.5–14.8)	9.1 (5.6–14.9)	0.959
Hemoglobin A1c, %	6.1 (5.9–6.3)	6.3 (6.1–6.4)	6.1 (5.8–6.4)	0.234
Total bilirubin, mg/dL	0.7 (0.6–1.0)	0.8 (0.7–1.0)	0.8 (0.7–1.0)	0.789
Serum albumin, g/dL	4.7 (4.6–4.8)	4.7 (4.5–4.8)	4.7 (4.6–4.9)	0.107
ALBI score	−3.28 (−3.39–−3.14)	−3.21 (−3.33–−3.06)	−3.21 (−3.38–−3.13)	0.091
M2BPGi	0.58 (0.49–0.77)	0.56 (0.39–0.76)	0.48 (0.42–0.69)	0.507

* Comparison between baseline and six months later using the Wilcoxon signed-rank test. IQR: interquartile range, AST: aspartate aminotransferase, ALT: alanine aminotransferase, γ-GTP: γ-glutamyl transpeptidase, GFR: glomerular filtration rate, LDL: low-density lipoprotein, HDL: high-density lipoprotein, ALBI: albumin–bilirubin, M2BPGi: Mac-2 binding protein glycosylation isomer.

## Data Availability

Data supporting the findings of this study are available from the corresponding author upon reasonable request.

## Supplementary Materials

The following supporting information can be downloaded from https://www.mdpi.com/article/10.3390/metabo15020100/s1: Figure S1. A waterfall plot showing changes in ALT levels from baseline to six months later.

## References

[1] Younossi Z., Tacke F., Arrese M., Chander Sharma B., Mostafa I., Bugianesi E., Wai-Sun Wong V., Yilmaz Y., George J., Fan J. (2019). Global Perspectives on Nonalcoholic Fatty Liver Disease and Nonalcoholic Steatohepatitis. Hepatology.

[2] Tokushige K., Ikejima K., Ono M., Eguchi Y., Kamada Y., Itoh Y., Akuta N., Iwasa M., Yoneda M., Otsuka M. (2021). Evidence-based clinical practice guidelines for nonalcoholic fatty liver disease/nonalcoholic steatohepatitis 2020. Hepatol. Res..

[3] Shinozaki S., Tahara T., Lefor A.K., Ogura M. (2020). Pemafibrate decreases markers of hepatic inflammation in patients with non-alcoholic fatty liver disease. Clin. Exp. Hepatol..

[4] Shinozaki S., Tahara T., Lefor A.K., Ogura M. (2021). Pemafibrate improves hepatic inflammation, function and fibrosis in patients with non-alcoholic fatty liver disease: A one-year observational study. Clin. Exp. Hepatol..

[5] Hassan M., Al-Obaidi H., Karrick M., Merza N., Nawras Y., Saab O., Al-Obaidi A.D., Merza F., Hashim H.T., Al Zubaidi K. (2024). Effect of Pemafibrate on the Lipid Profile, Liver Function, and Liver Fibrosis Among Patients With Metabolic Dysfunction-Associated Steatotic Liver Disease. Gastroenterol. Res..

[6] Nakajima A., Eguchi Y., Yoneda M., Imajo K., Tamaki N., Suganami H., Nojima T., Tanigawa R., Iizuka M., Iida Y. (2021). Randomised clinical trial: Pemafibrate, a novel selective peroxisome proliferator-activated receptor α modulator (SPPARMα), versus placebo in patients with non-alcoholic fatty liver disease. Aliment. Pharmacol. Ther..

[7] Nakajima A., Eguchi Y., Yoneda M., Imajo K., Tamaki N., Suganami H., Nojima T., Tanigawa R., Iizuka M., Iida Y. (2022). Triglyceride Lowering with Pemafibrate to Reduce Cardiovascular Risk. N. Engl. J. Med..

[8] Rinella M.E., Lazarus J.V., Ratziu V., Francque S.M., Sanyal A.J., Kanwal F., Romero D., Abdelmalek M.F., Anstee Q.M., Arab J.P. (2023). A multisociety Delphi consensus statement on new fatty liver disease nomenclature. Hepatology.

[9] Rinella M.E., Neuschwander-Tetri B.A., Siddiqui M.S., Abdelmalek M.F., Caldwell S., Barb D., Kleiner D.E., Loomba R. (2023). AASLD Practice Guidance on the clinical assessment and management of nonalcoholic fatty liver disease. Hepatology.

[10] Hiraoka A., Kumada T., Kudo M., Hirooka M., Tsuji K., Itobayashi E., Kariyama K., Ishikawa T., Tajiri K., Ochi H. (2017). Albumin-Bilirubin (ALBI) Grade as Part of the Evidence-Based Clinical Practice Guideline for HCC of the Japan Society of Hepatology: A Comparison with the Liver Damage and Child-Pugh Classifications. Liver Cancer.

[11] Abe M., Miyake T., Kuno A., Imai Y., Sawai Y., Hino K., Hara Y., Hige S., Sakamoto M., Yamada G. (2015). Association between Wisteria floribunda agglutinin-positive Mac-2 binding protein and the fibrosis stage of non-alcoholic fatty liver disease. J. Gastroenterol..

[12] Shinozaki S., Tahara T., Miura K., Lefor A.K., Yamamoto H. (2022). Pemafibrate therapy for non-alcoholic fatty liver disease is more effective in lean patients than obese patients. Clin. Exp. Hepatol..

[13] Sasaki Y., Asahiyama M., Tanaka T., Yamamoto S., Murakami K., Kamiya W., Matsumura Y., Osawa T., Anai M., Fruchart J.C. (2020). Pemafibrate, a selective PPARα modulator, prevents non-alcoholic steatohepatitis development without reducing the hepatic triglyceride content. Sci. Rep..

[14] Choe H.J., Moon J.H., Kim W., Koo B.K., Cho N.H. (2024). Steatotic liver disease predicts cardiovascular disease and advanced liver fibrosis: A community-dwelling cohort study with 20-year follow-up. Metabolism.

[15] Ong Lopez A.M.C., Pajimna J.A.T. (2024). Efficacy of sodium glucose cotransporter 2 inhibitors on hepatic fibrosis and steatosis in non-alcoholic fatty liver disease: An updated systematic review and meta-analysis. Sci. Rep..

[16] Shinozaki S., Tahara T., Miura K., Lefor A.K., Yamamoto H. (2023). Effectiveness of One-Year Pemafibrate Therapy on Non-Alcoholic Fatty Liver Disease Refractory to Long-Term Sodium Glucose Cotransporter-2 Inhibitor Therapy: A Pilot Study. Life.

[17] Yokote K., Yamashita S., Arai H., Araki E., Matsushita M., Nojima T., Suganami H., Ishibashi S. (2021). Effects of pemafibrate on glucose metabolism markers and liver function tests in patients with hypertriglyceridemia: A pooled analysis of six phase 2 and phase 3 randomized double-blind placebo-controlled clinical trials. Cardiovasc. Diabetol..

