# Supplementary material for: Effectiveness of Pemafibrate Dose Escalation on Metabolic Dysfunction-Associated Steatotic Liver Disease Refractory to Standard Dose
Source: Metabolites. 2025 Feb 5;15(2):100. doi: 10.3390/metabo15020100 (PMC11857616; doi:10.3390/metabo15020100)
Supplement: Supplementary file 1 [file metabolites-15-00100-s001.zip › metabolites-3452336-supplementary.pdf]

Supplementary Materials

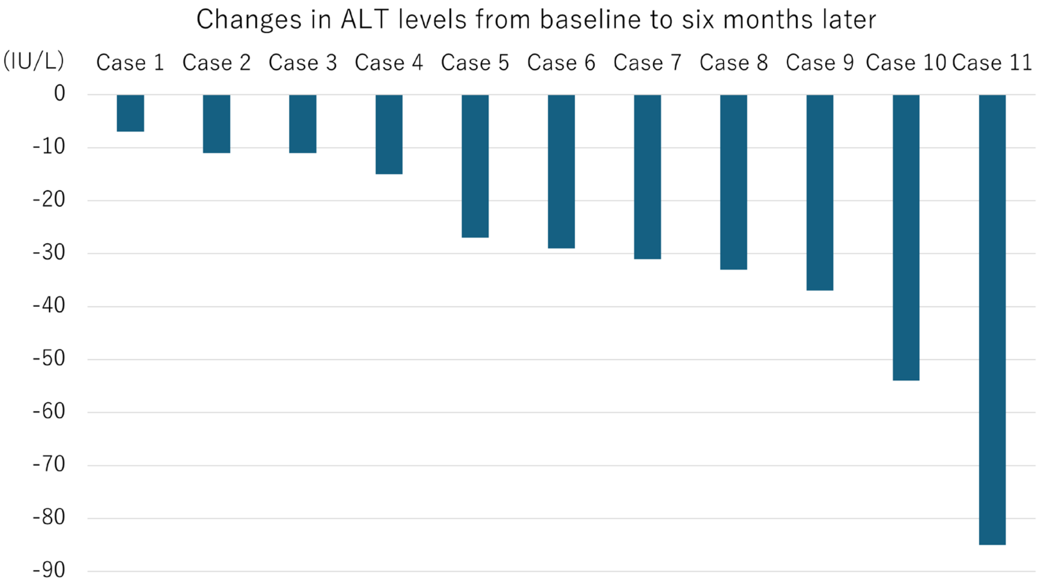

Figure S1. A waterfall plot showing changes in ALT levels from baseline to six months later.
